# Three new species of *Fonsecaiulus* (Hemiptera, Cicadellidae, Cicadellini) from Brazil and key to species of the genus

**DOI:** 10.3897/zookeys.526.6154

**Published:** 2015-10-12

**Authors:** Márcio Felix, Cauan Antunes, Rachel A. Carvalho, Gabriel Mejdalani

**Affiliations:** 1 Laboratório de Biodiversidade Entomológica, Instituto Oswaldo Cruz, Fundação Oswaldo Cruz, Av. Brasil 4365, 21040-360, Rio de Janeiro, RJ, Brasil; 2 Departamento de Entomologia, Museu Nacional, Universidade Federal do Rio de Janeiro, Quinta da Boa Vista, São Cristóvão, 20940-040, Rio de Janeiro, RJ, Brasil

**Keywords:** Atlantic Forest, Auchenorrhyncha, Cerrado, leafhopper, morphology, sharpshooter, taxonomy

## Abstract

Three new sharpshooter species of the genus *Fonsecaiulus* Young, 1977 are described and illustrated from specimens collected in the Brazilian Atlantic Forest, *Fonsecaiulus rectangularis* and *Fonsecaiulus guttiformis*, and in the Brazilian Cerrado, *Fonsecaiulus filiformis*. The descriptions are based on features from the external morphology, color pattern, and male and female genital structures. Comparisons of the three new taxa with the remaining six *Fonsecaiulus* species are provided. An identification key to males of all known species of the genus is given.

## Introduction

The genus *Fonsecaiulus* Young, 1977 occurs in Venezuela, NE, CW, SE and S Brazil, Bolivia, and Argentina, being composed of six species (Young 1977): *Fonsecaiulus cognatus* (Schmidt, 1928); *Fonsecaiulus dorsifascia* (Osborn, 1926); *Fonsecaiulus flavovittata* (Stål, 1859), the type species; *Fonsecaiulus gaudialis* Young, 1977; *Fonsecaiulus sanguineovittata* (Signoret, 1855); and *Fonsecaiulus sciotus* Young, 1977. Specimens of *Fonsecaiulus* have a conspicuous median yellow stripe covering at least the anterior dorsum, limited by a pair of black to brown stripes or areas.

In this paper three new species of *Fonsecaiulus* are described and illustrated from specimens collected in Atlantic Forest areas from Espírito Santo State, SE. Brazil, and in the Cerrado (tropical savanna) from Goiás State, CW. Brazil. An identification key to males of all known species of the genus is given. Notes comparing the three new taxa with the remaining *Fonsecaiulus* species are provided.

## Material and methods

The genital structures were prepared according to the techniques of Oman (1949) and Mejdalani (1998) for males and females, respectively. The dissected parts were stored in small vials with glycerin and attached below the specimens. Morphological terminology follows mainly Young (1977), except for the head (Hamilton 1981, Mejdalani 1993, 1998) and the female genitalia (Hill 1970, Davis 1975).

The specimens herein studied were deposited in the Coleção Entomológica do Instituto Oswaldo Cruz, Fundação Oswaldo Cruz (CEIOC, Rio de Janeiro), Coleção Entomológica Prof. José Alfredo P. Dutra, Departamento de Zoologia, Instituto de Biologia, Universidade Federal do Rio de Janeiro (DZRJ, Rio de Janeiro), and Departamento de Entomologia, Museu Nacional, Universidade Federal do Rio de Janeiro (MNRJ, Rio de Janeiro). Label data of type specimens are given inside quotations with a reversed virgule [\] separating lines on a label and a semicolon separating different labels.

## Taxonomy

### Fonsecaiulus rectangularis sp. n.

Taxon classificationAnimaliaHemipteraCicadellidae

http://zoobank.org/40A8F229-4B6A-4139-9A0E-8C80C64066F4

Fig. 1

#### Diagnosis.

**Figure 1.**
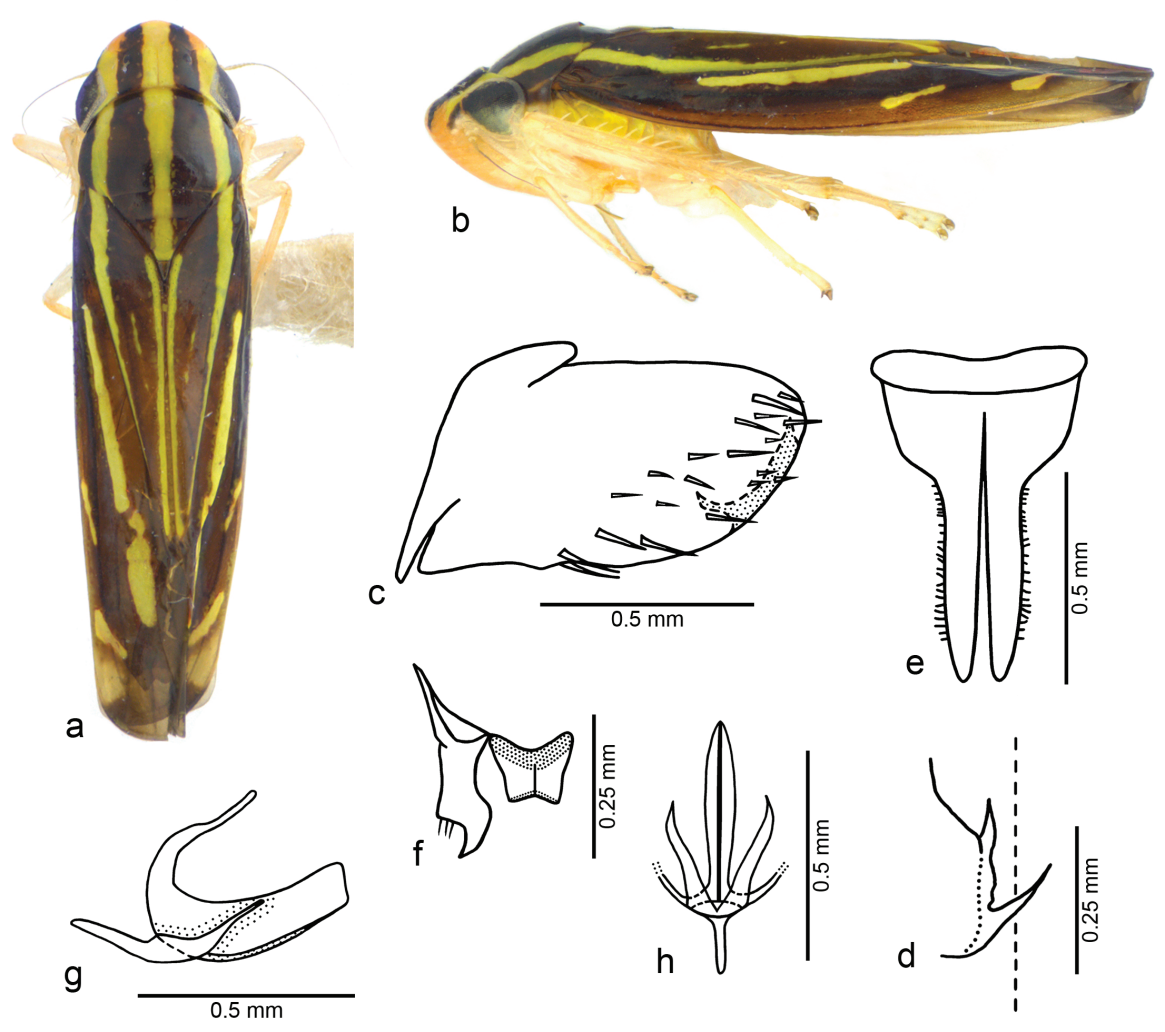
*Fonsecaiulus rectangularis* sp. n., male holotype. **a** body, dorsal view **b** body, lateral view **c** pygofer, lateral view **d** apical left portion of pygofer, posterior view **e** valve and subgenital plates, ventral view **f** left style and connective, dorsal view **g** aedeagus and paraphyses, lateral view **h** aedeagus and paraphyses, ventral view. Body length: 5.5 mm.

*Fonsecaiulus rectangularis* sp. n. is characterized by the combination of the following features: (1) male pygofer with two acute processes (Fig. 1d), one posterodorsal, short and spiniform, and another posteroventral, long; (2) aedeagus with shaft long and moderately broad in lateral view (Fig. 1g), without processes, apex truncate to slightly concave; (3) paraphyses (Fig. 1h) with pair of simple long rami.

#### Etymology.

The specific epithet, *rectangularis*, refers to the shape of the aedeagal shaft in lateral view.

#### Description.

Length. Male holotype, 5.5 mm; male paratypes, 5.3–5.5 mm.

Male holotype. Head and thorax. Head (Fig. 1a, b) with median length of crown 7/10 interocular width and 4/10 transocular width; frons not flattened medially, muscle impressions distinct; epistomal suture obsolete medially; clypeus with contour continuing profile of frons. Pronotum (Fig. 1a, b) with width equal to transocular width; lateral margins slightly convergent anteriorly. Remaining morphological characteristics of head and thorax as in the generic description of Young (1977: 760–763).

Male genitalia. Pygofer (Fig. 1c, d) with dorsoapical short spine, directed dorsally; ventroapical margin with inner long process, anteromedially turned. Valve (Fig. 1e) short and broad, anterior margin concave medially and posterior margin convex. Subgenital plates (Fig. 1e) narrow on apical two-thirds; dorsal surface with two minute, sclerotized dentiform processes on median portion, near which apical portion of styles rests; short microsetae along outer margin. Styles (Fig. 1f) with outer preapical portion with long sparse setae; apex narrowly truncate. Connective (Fig. 1f) short and broad in dorsal view, with median keel. Aedeagus (Fig. 1g, h), in lateral view, with shaft broad and elongate, curved dorsally, subrectangular; apex truncate; ventral margin laterally expanded in caudal view; dorsal apodemes long and curved posteriorly. Paraphyses (Fig. 1g, h) symmetrical, with pair of long rami extending as far posteriorly as half of aedeagal shaft, posteriorly divergent in ventral view; rami curved dorsally, with apical half dorsoventrally flattened, apex acute.

Color. Dorsum brown with three longitudinal yellow stripes (Fig. 1a, b); median stripe extending from apex of crown to apex of clavus, posteriorly narrowed from median portion of pronotum; pair of lateral stripes extending posteriorly from frontogenal suture along claval sulcus, almost attaining its apex, strongly narrowed on posterior two-thirds of sulcus. Crown (Fig. 1a, b) with lateral areas anteriorly to frontogenal sutures pale orange. Clavus (Fig. 1a, b) with narrow, median, elongate oblique yellow macula. Corium (Fig. 1a, b) with yellow stripe parallel and adjacent to median portion of brachial cell; yellow elongate macula on inner anteapical cell; two smaller oblique yellow maculae near costal margin, anterior one opposite claval apex, posterior one on outer anteapical cell. Face pale orange. Frons with pair of dorsolateral brown maculae continuous with color pattern of crown. Antennal ledges brown (Fig. 1b). Thoracic sclerites mostly yellow (Fig. 1b); lateral lobe of pronotum dorsally brown. Legs mostly pale orange (Fig. 1b). Thoracic sternum mostly pale orange.

Female unknown.

#### Intraspecific variation

(based on eight male paratypes). The direction of the pygofer processes is variable; the aedeagal shaft can be more dorsally curved than in the holotype; its apical portion, in lateral view, can be broader and the apical margin, slightly concave.

#### Type specimens.

Brazil, Espírito Santo State. Holotype: male, “BR, ES, Sta. Teresa, Est. \ Biol. Santa Lúcia, 16.V.2012, \ Buys, Leibão & Antunes \ leg.” (CEIOC). Paratypes: two males, same data as holotype (CEIOC); four males, “BR, ES, Sta. Teresa, Est. \ Biol. Santa Lúcia, 18.X.2012, \ Buys, Cordeiro & Tinoco, \ leg. Prato amarelo” (CEIOC); two males, “BR, ES, Santa Maria de \ Jetibá, Fazenda Azaléia, \ 18.V.2012, Buys, Leibão & \ Antunes leg.” (MNRJ).

#### Remarks.

*Fonsecaiulus rectangularis* sp. n. (Fig. 1a, b) is similar in color and distributional pattern of stripes to *Fonsecaiulus flavovittata* and *Fonsecaiulus gaudialis*. The pair of yellow stripes extending posteriorly from the frontogenal sutures is narrower than in *Fonsecaiulus flavovittata* on anterior portion of claval sulcus (Wilson et al. 2009: http://naturalhistory.museumwales.ac.uk/sharpshooters/browserecord.php?-recid=1012).

The male genital structures are similar to those of *Fonsecaiulus gaudialis*. The posterior margin of the pygofer presents acute processes in both species, being a single ventral process in *Fonsecaiulus gaudialis*, not attaining the median line (Young 1977: figs 627c, p). In the new species, there are two processes: one dorsal, short and spiniform, and another ventral, long (Fig. 1c, d). The aedeagal shaft in both species is long and moderately broad in lateral view, without processes. The shaft apex is truncate to slightly concave in *Fonsecaiulus rectangularis* (Fig. 1g), while it is convex in *Fonsecaiulus gaudialis* (Young 1977: fig. 627f). The paraphyses have a pair of simple long rami in the new species (Fig. 1h). In *Fonsecaiulus gaudialis* each ramus is clearly bifid (Young 1977: fig. 627h).

### Fonsecaiulus guttiformis sp. n.

Taxon classificationAnimaliaHemipteraCicadellidae

http://zoobank.org/D23721F8-37E9-4E6E-B444-60300CD6804C

Figs 2 , 3

#### Diagnosis.

**Figure 2.**
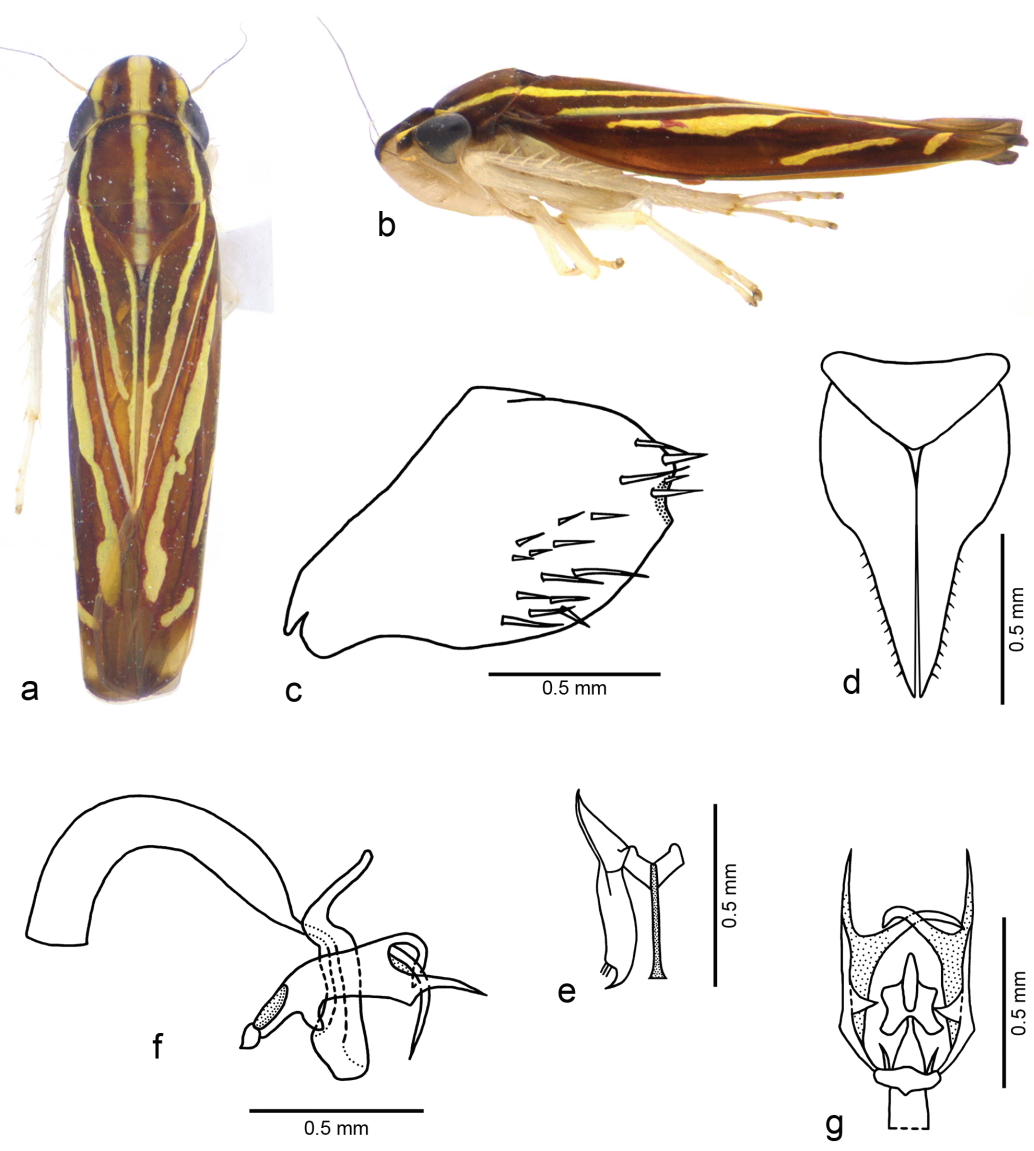
*Fonsecaiulus guttiformis* sp. n., male holotype. **a** body, dorsal view **b** body, lateral view **c** pygofer, lateral view **d** valve and subgenital plates, ventral view **e** left style and connective, dorsal view **f** ejaculatory reservoir, aedeagus, and paraphyses, lateral view **g** part of ejaculatory reservoir, aedeagus, and paraphyses, ventral view. Body length: 5.4 mm.

**Figure 3.**
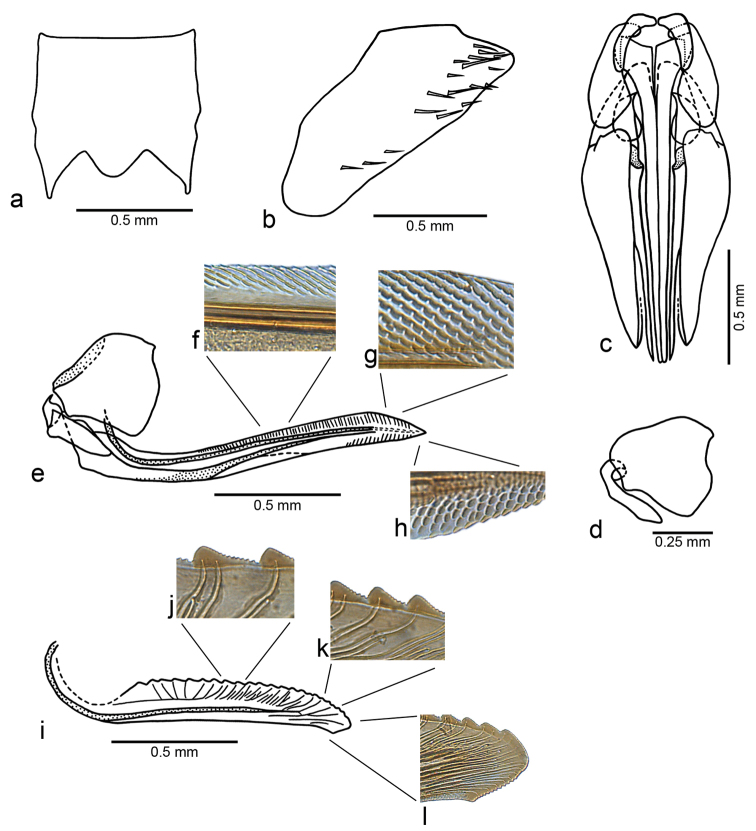
*Fonsecaiulus guttiformis* sp. n., female paratype. **a** abdominal sternite VII, ventral view **b** pygofer, lateral view **c** apical portion of abdomen with sternite VII removed (macrosetae of pygofer omitted), ventral view **d** first valvifer, lateral view **e** first valvifer and valvula of ovipositor, lateral view **f** basal and **g** apical dorsal sculpturing in detail **h** apical ventral sculpturing in detail **i** second valvula of ovipositor, lateral view **j** median **k** preapical, and **l** apical teeth and denticles in detail.

*Fonsecaiulus guttiformis* sp. n. is characterized by the combination of the following features: (1) single yellow stripe on median portion of clavus (Fig. 2a), directed to commissural margin; (2) valve (Fig. 2d) broad and subtriangular; (3) styles and connective stalk (Fig. 2e) very elongate; (4) aedeagus (Fig. 2f) strongly curved ventrally with apex broad; (5) paraphyses (Fig. 2f, g) very complex, with short basal plate and pair of broad and long rami with processes; (6) female sternite VII (Fig. 3a) subtriangularly produced posterolaterally, with well-produced median lobe.

#### Etymology.

The specific epithet, *guttiformis*, refers to the shape of the aedeagal shaft in lateral view.

#### Description.

Length. Male holotype, 5.4 mm; male paratypes, 5.0–5.5 mm; female paratype, 5.5 mm.

Male holotype. Head and thorax. Head (Fig. 2a, b) with median length of crown slightly less than 7/10 interocular width and slightly less than 4/10 transocular width; frons slightly flattened medially, muscle impressions distinct; epistomal suture obsolete medially; clypeus with contour continuing profile of frons. Pronotum (Fig. 2a, b) with width equal to transocular width; lateral margins slightly convergent anteriorly. Remaining morphological characteristics of head and thorax as in the generic description of Young (1977: 760–763).

Male genitalia. Pygofer (Fig. 2c) slightly concave posteriorly; ventroapical margin with small rounded lobe, directed medially. Valve (Fig. 2d) broad and subtriangular. Subgenital plates (Fig. 2d) narrow on apical half; dorsal surface with two minute, sclerotized dentiform processes on median portion, near which apical portion of styles rests; short microsetae along outer margin. Styles (Fig. 2e) elongate, extending as far posteriorly as connective apex; outer preapical portion with long sparse setae; apex directed outwards. Connective (Fig. 2e) Y-shaped in dorsal view; stalk elongate, with well-produced median keel. Aedeagus (Fig. 2f, g), in lateral view, with shaft long and gutiform, strongly curved ventrally; apex broadly convex; gonopore apical; dorsal apodemes long and curved posteriorly. Paraphyses (Fig. 2f, g) symmetrical, with short basal plate and pair of complex broad and long rami; each ramus with inner basal process, slender and very short; ventral margin with short process between basal and median thirds, slightly curved posteriorly; apex bifurcated into two long and narrow acute processes, inner one posteromedially curved and crossing median line of pygofer, the other one directed posteriorly, with short triangular basiventral projection.

Color. Dorsum brown with longitudinal yellow stripes (Fig. 2a, b). Head and thorax (Fig. 2a, b) with three stripes, median one extending from apex of crown to apex of clavus, posteriorly narrowed from median portion of pronotum, and pair of lateral stripes extending from frontogenal suture to median portion of clavus, almost attaining median portion of commissural margin. Clavus (Fig. 2a, b) with narrow yellow stripe adjacent to claval sulcus, absent on basal portion. Corium (Fig. 2a, b) with broad yellow irregular stripe adjacent to brachial cell, extending posteriorly to inner anteapical cell, narrowed on portion opposite claval apex; two elongate oblique yellow maculae near costal margin, anterior one opposite claval apex (interrupted in the right forewing) and posterior one on outer anteapical cell. Face pale yellow. Frons with pair of dorsolateral brown maculae continuous with color pattern of crown. Antennal ledges brown (Fig. 2b). Thoracic sclerites (Fig. 2b) mostly yellow; lateral lobe of pronotum dorsally brown. Legs (Fig. 2b) mostly pale yellow. Thoracic sternum mostly pale yellow.

Female genitalia (based on one paratype). Sternite VII (Fig. 3a) subtriangularly produced posterolaterally; posterior margin with well-produced median lobe. “Internal” sternite VIII without sclerites. Pygofer (Fig. 3b, c) moderately produced posteriorly in lateral view; surface with sparse row of macrosetae along ventroapical margin and a few grouped near apex. First valvifers (Fig. 3c–e) large, subrectangular in lateral view, each with long, basally articulated anterior process directed posteroventrally; basal portion of processes, in ventral view, medially produced and connected to each other by membrane (Fig. 3c). First ovipositor valvulae (Fig. 3e–h) with basal portion enlarged and subrectangular; basal margin truncate and oblique in ventral view (Fig. 3c); sculptured areas mostly scalelike, with linear tegumentary processes on basidorsal portion (Fig. 3f) and separated scales on ventroapical portion (Fig. 3h); ventral margin broadly concave; apex acute. Second valvulae (Fig. 3i–l) broadened beyond basal curvature, narrowing slightly towards narrowly rounded apex; ventral margin approximately rectilinear; preapical prominence (Fig. 3l) conspicuous, narrowly rounded; dorsal margin with approximately 22 mostly triangular continuous teeth, extending from expanded basal portion to apical portion of blade; most teeth with steep, small ascending portion, and gradually declivous, large descending portion (Fig. 3j, k); denticles distributed on teeth (Fig. 3j, k) and on apical portion of blade, except on apex (Fig. 3l); blade with ducts attaining teeth or terminating below them, also extending to apex (Fig. 3i–l). Gonoplacs with basal half distinctly narrow, abruptly expanded on median portion; ventral margin slightly concave on median third; apex rounded.

Intraspecific variation (based on nine male and one female paratypes). Short curved process between basal and median third of paraphyses rami with variable length; ventral margin of each ramus sometimes irregular, with slight projections and emarginations.

#### Type specimens.

Brazil, Espírito Santo State. Holotype: male, “Coleção Santa \ Teresa”; “BR, ES, Sta. Teresa, Est. \ Biol. Santa Lúcia 17- \ 21.IV.2012, Buys & Leibão \ leg.” (CEIOC). Paratypes: one male and one female, same data as holotype (CEIOC); three males, “BR, ES, Sta. Teresa, Est. \ Biol. Santa Lúcia, Trilha do \ Ruschi, 22.VII.2012, Buys, \ leg. Prato Amarelo” (CEIOC); one male, “BR, ES, Sta. Teresa, Est. \ Biol. Santa Lúcia, 18.X.2012, \ Buys, Cordeiro & Tinoco, \ leg. Prato amarelo” (MNRJ); four males, “BR, ES, Sta. Teresa, Est. \ Biol. Santa Lúcia, Trilha do \ Rio, 17.X.2012, Buys, \ Cordeiro & Tinoco leg.” (CEIOC).

#### Remarks.

*Fonsecaiulus guttiformis* sp. n. (Fig. 2a, b) is similar in color pattern and male and female structures to *Fonsecaiulus cognatus*. In the new species the lateral yellow stripes on anterior dorsum converge posteriorly to the commissural claval margins (Fig. 2a). In *Fonsecaiulus cognatus* these stripes have similar position on clavus but they are paired (Wilson et al. 2009: http://naturalhistory.museumwales.ac.uk/sharpshooters/browserecord.php?-recid=1008).

The male genitalia of *Fonsecaiulus guttiformis* are the most distinct in the genus. The valve is broad and subtriangular (Fig. 2d), whereas this structure is short and broadly convex posteriorly in the remaining species of the genus. The styles and connective stalk are uncommonly elongate (Fig. 2e). The aedeagus is strongly curved ventrally with the apex broad (Fig. 2f). *Fonsecaiulus cognatus* is the only other known species in which the aedeagal shaft has a ventral curvature (Young 1977: fig. 625q), but it is slighter than in *Fonsecaiulus guttiformis*. The paraphyses are very complex in the latter species, with short basal plate and pair of broad and long rami presenting processes (Fig. 2f, g). Until now, the paraphyses of *Fonsecaiulus flavovittata* were the most complex in the genus (Young 1977: fig. 622r).

Regarding the female genitalia, the sternite VII of *Fonsecaiulus guttiformis* (Fig. 3a) is similar to that of *Fonsecaiulus cognatus* (Young 1977: fig. 625i), both being posterolaterally produced and with a well-produced median lobe. The lateral lobes in the new species are subtriangular, whereas in *Fonsecaiulus cognatus* they are narrowly rounded.

The first valvifers of *Fonsecaiulus guttiformis* bear a conspicuous anterior process that is basally articulated (Fig. 3d, e). Young (1977) described a pair of elongate processes projecting from the dorsal membrane into the genital chamber in *Fonsecaiulus sciotus* (see fig. 626p from that author). The position and shape of these processes are similar to the ones observed in *Fonsecaiulus guttiformis*. Carvalho and Mejdalani (2014) described processes originating from the same portion of the valvifers, but not basally articulated to them, in two species of *Erythrogonia* Melichar, 1926: *Erythrogonia phoenicea* (Signoret, 1853) (see fig. 8 from those authors) and *Erythrogonia calva* (Taschenberg, 1884) (see fig. 22 from those authors). This genus, as well as *Fonsecaiulus*, is included in the *Erythrogonia* generic group (Young 1977).

### Fonsecaiulus filiformis sp. n.

Taxon classificationAnimaliaHemipteraCicadellidae

http://zoobank.org/D600EBDB-6855-4C5A-9B9A-7D3AE05C14F4

Fig. 4

#### Diagnosis.

**Figure 4.**
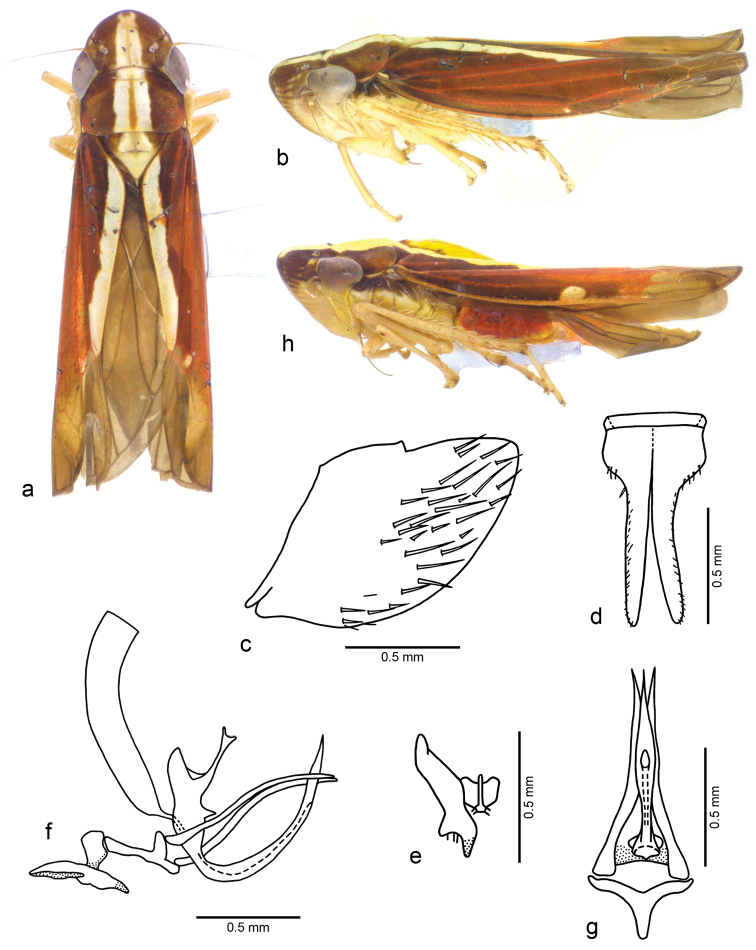
*Fonsecaiulus filiformis* sp. n., male holotype. **a** body, dorsal view **b** body, lateral view **c** pygofer, lateral view **d** valve and subgenital plates, ventral view **e** left style and connective, dorsal view **f** left style, connective, ejaculatory reservoir, aedeagus, and paraphyses, lateral view **g** aedeagus and paraphyses, ventral view. *Fonsecaiulus dorsifascia* (Osborn, 1926) **h** body, lateral view. Body length of *Fonsecaiulus filiformis* 5.6 mm and of *Fonsecaiulus dorsifascia* 5.7 mm.

*Fonsecaiulus filiformis* sp. n. is characterized by the combination of the following features: (1) dorsum (Fig. 4a) with broad pale yellow median stripe extending from apex of crown to apex of clavus; (2) connective (Fig. 4e, f) with median keel strongly produced dorsally; (3) aedeagus (Fig. 4f) with shaft long and slender, dorsally curved, with long and acute apical process continuing its shape; (4) paraphyses (Fig. 4g) with Y-shaped basal plate with arms widely divergent and pair of long and slender rami.

#### Etymology.

The specific epithet, *filiformis*, refers to the shape of the aedeagus in lateral view.

#### Description.

Length. Male holotype, 5.6 mm.

Male holotype. Head and thorax. Head (Fig. 4a, b) with median length of crown slightly less than 7/10 interocular width and slightly less than 4/10 transocular width; ocelli located slightly behind a line between anterior eye angles; frons slightly flattened medially, muscle impressions distinct; epistomal suture complete; clypeus with contour continuing profile of frons. Pronotum (Fig. 4a, b) with width less than transocular width; lateral margins parallel. Forewings with inner and median anteapical cells opened basally. First tarsomere (Fig. 4b) with length approximately equal to combined length of two more distal tarsomeres. Remaining morphological characteristics of head and thorax as in the generic description of Young (1977: 760–763).

Male genitalia. Pygofer (Fig. 4c) broadly convex posteriorly, posterodorsal portion slightly produced, without processes. Valve (Fig. 4d) broad and very short, subrectangular. Subgenital plates (Fig. 4d) narrow on apical three-fourths; dorsal surface with two minute, sclerotized dentiform processes on median portion, near which apical portion of styles rests; basal portion with few short macrosetae along outer margin; few very short microsetae on apical half. Styles (Fig. 4e, f) with outer preapical portion with long sparse setae; apex directed outwards. Connective (Fig. 4e, f) with arms short in dorsal view; stalk moderately elongate, with strongly produced median keel. Aedeagus (Fig. 4f, g), in lateral view, with shaft long and slender, dorsally curved; apex with long and acute process continuing shape of shaft, extending dorsally beyond pygofer border; gonopore ventral; dorsal apodemes long and slightly curved posteriorly. Paraphyses (Fig. 4f, g) symmetrical, basal plate Y-shaped and arms widely divergent; rami articulated to basal plate, long and slender, with apex acute, extending posteriorly beyond pygofer border; each ramus, in lateral view, sinuous, slightly curved dorsally and then slightly curved ventrally.

Color. Dorsum anteriorly brown to yellowish-brown with broad pale yellow median stripe extending from apex of crown to apex of clavus (Fig. 4a, b); stripe occupying approximately one-third of posterior margin of crown, with lateral margins sinuous on clavus. Crown (Fig. 4a) with pair of small pale yellow spots on antennal ledges and another pair adjacent to outer margin of ocelli; subtriangular pale yellow marks adjacent to inner eye angles. Pronotum (Fig. 4a, b) with narrow, median yellowish-brown stripe on pale yellow broad stripe; humeral areas reddish-brown. Forewings (Fig. 4a, b) with basal portion dark brown, median portion and most of corium veins reddish-brown, apical portion pale brown; minute pale yellow spot beyond middle of costal margin and another on apex of brachial cell. Face pale yellow. Frons with median portion bordered by pair of longitudinal narrow brown stripes, connected to each other on dorsal median portion of clypeus, then extending ventrally as a median stripe; narrow transverse brown stripes along muscle impressions. Antennal ledges (Fig. 4b), in lateral view, almost entirely brown. Thoracic sclerites (Fig. 4b) mostly pale yellow; lateral lobe of pronotum, mesepimeron, and mesepisternum dorsally dark brown. Legs (Fig. 4b) mostly pale yellow. Thoracic sternum mostly pale yellow.

Female unknown.

#### Type specimen.

Brazil, Goiás State. Holotype: male, “Brasil, GO, Alto Paraíso \ de Goiás, Parque \ Nacional da Chapada dos \ Veadeiros, trilha para \ canion”; “S14°10'5", W47°49'16" \ 941m 25.X.2013 sweep \ DM Takiya, BM Camisão \ e CC Gonçalves leg.” (DZRJ).

#### Remarks.

*Fonsecaiulus filiformis* sp. n. (Fig. 4a, b) is very similar to *Fonsecaiulus dorsifascia* (Fig. 4h) in color pattern and male structures. Only these two species have a broad, median pale yellow stripe on dorsum. In the new species the lateral margins of the stripe are sinuous on forewings, whereas in *Fonsecaiulus dorsifascia* they are triangularly emarginated.

In the male genitalia, the pygofer and subgenital plates are very similar in both species. The apical portion of the styles in *Fonsecaiulus filiformis* differs from that of *Fonsecaiulus dorsifascia*. In the former species, the preapical lobe is more produced (Fig. 4e) than in the latter (Young 1977: fig. 624e). The paraphyses of the new species are similar to those of *Fonsecaiulus dorsifascia*. The aedeagi are strongly curved dorsally in both species. However, *Fonsecaiulus filiformis* has a regularly very narrow aedeagus in lateral view (Fig. 4f), while *Fonsecaiulus dorsifascia* has the aedeagal shaft broader (Young 1977: fig. 624f).

### Additional material of *Fonsecaiulus* examined

*Fonsecaiulus dorsifascia* – Brazil – Goiás State: one male, Alto Paraíso de Goiás (DZRJ).

*Fonsecaiulus flavovittata* – Brazil – Espírito Santo State: seven males, Santa Teresa (CEIOC); one male, Santa Maria de Jetibá (CEIOC).

### Key to males of *Fonsecaiulus*

**Table:** 

1	Mesonotum dark brown, rarely with small faint pale yellow marks anteriorly; paraphyses with pair of long and narrow rami, each with short process on median portion and a shorter one on apical portion (Young 1977: fig. 626h)	***Fonsecaiulus sciotus***
–	Mesonotum with distinct longitudinal yellow stripes; paraphyses with rami not as above	**2**
2	Dorsum with a single broad yellow stripe extending from anterior margin of crown to claval apex (Fig. 4a)	**3**
–	Dorsum with some yellow stripes, generally narrow (Figs 1a, 2a)	**4**
3	Dorsal yellow stripe broad and occupying most of claval region (Fig. 4h) and with outer boarder markedly serrated on clavus; costal yellow mark large and rounded (Fig. 4h); aedeagus, in lateral view, with shaft moderately broad with long dorsoapical acute process (Young 1977: fig. 624f)	***Fonsecaiulus dorsifascia***
–	Dorsal yellow stripe narrower and not occupying most of claval region (Fig. 4a, b) and with outer boarder slightly sinuous on clavus; costal yellow mark a very small dot (Fig. 4b); aedeagus, in lateral view, with shaft slender with long dorsoapical acute process continuing its shape (Fig. 4f)	***Fonsecaiulus filiformis* sp. n.**
4	Crown with median yellow stripe much broader than the adjacent brown stripes	**5**
–	Crown with median yellow stripe approximately as broad as (Fig. 1a) or narrower than (Fig. 2a) the adjacent brown stripes	**6**
5	Clavus with two oblique yellow stripes on central portion, posteriorly directed to commissural margin; aedeagus ventrally curved (Young 1977: fig. 625q); paraphyses with rami crossing each other (Young 1977: fig. 625q, r)	***Fonsecaiulus cognatus***
–	Clavus with one oblique yellow stripe on central portion, posteriorly directed to commissural margin; aedeagus dorsally curved (Young 1977: fig. 623f); paraphyses with rami posteriorly divergent (Young 1977: fig. 623p)	***Fonsecaiulus sanguineovittata***
6	Aedeagus, in lateral view, with shaft strongly narrowed towards apex (Young 1977: fig. 622f)	***Fonsecaiulus flavovittata***
–	Aedeagus, in lateral view, with shaft broad	**7**
7	Pygofer without acute processes (Fig. 2c); subgenital plates narrowed on apical half (Fig. 2d); aedeagus strongly curved ventrally (Fig. 2f)	***Fonsecaiulus guttiformis* sp. n.**
–	Pygofer with at least one acute process; subgenital plates narrowed on apical two-thirds; aedeagus dorsally curved	**8**
8	Pygofer with single apical process (Young 1977: fig. 627c, p); aedeagus with apex convex in lateral view (Young 1977: fig. 627f); paraphyses with pair of long bifid rami (Young 1977: fig. 627h)	***Fonsecaiulus gaudialis***
–	Pygofer with two apical processes (Fig. 1c, d); aedeagus with apex truncate to slightly concave in lateral view (Fig. 1g); paraphyses with pair of long simple rami (Fig. 1g, h)	***Fonsecaiulus rectangularis* sp. n.**

## Supplementary Material

XML Treatment for Fonsecaiulus rectangularis

XML Treatment for Fonsecaiulus guttiformis

XML Treatment for Fonsecaiulus filiformis
